# Histological In Vivo Evaluation of Intense Pulsed Light Technology: Assessing the Safety on Oral Soft and Hard Tissues

**DOI:** 10.3390/dj12060151

**Published:** 2024-05-21

**Authors:** Yaniv Mayer, Amit Shenhav, Amin Younis, Eran Gabay, Hadar Giladi Zigdon

**Affiliations:** 1 Department of Periodontology, School of Graduate Dentistry, Rambam Health Care Campus, Haifa 3525408, Israel; 2 The Ruth and Bruce Rappaport Faculty of Medicine, Technion, Haifa 3200003, Israel; 3 Laboratory for Bone Repair, Rambam Health Care Campus, Haifa 3525408, Israel

**Keywords:** intense pulsed light, oral biopsy, thermal damage, histological response

## Abstract

Intense pulsed light (IPL) is used for aesthetic and therapeutic purposes. According to recent literature, utilizing IPL may boost upregulation of anti-inflammatory cytokines, and downregulation of pro-inflammatory cytokines. Concerns have been raised about potential thermal damage to the soft and hard tissues in the oral cavity. Therefore, the aim of this study was to determine the safety of using IPL of various intensities in the tissues of the oral cavity. Methods: Three adult pigs were included in the trial. The oral cavity was divided into four quadrants and projected with a wide range of IPL settings. Alveolar bone, buccal mucosa, and gingival tissue samples were taken immediately and after 24 h. In each animal, one quadrant of the jaw was left untreated and served as a control. All samples were processed and stained with H&E. Results: Clinical examination showed no evidence of changes in the integrity of the examined tissues. Histological examination of the different tissues did not demonstrate significant thermal damage or changes in the characterization of the cells compared to the control tissues. Conclusions: The use of IPL in the oral cavity is safe and does not negatively affect the tissues.

## 1. Introduction

Intense pulsed light (IPL) is a technology used by cosmetic and medical practitioners to perform various skin treatments for aesthetic and therapeutic purposes, including hair removal and photo rejuvenation, as well as to alleviate dermatologic diseases, such as acne [1,2]. IPL is also being increasingly used in optometry and ophthalmology, to treat evaporative dry-eye disease due to meibomian gland dysfunction [3].

IPL uses a flashlamp to emit polychromatic light across a wide wavelength spectrum of approximately 400 to 1200 nm. Both lasers and light devices, such as IPL, produce a clinical effect when their light is absorbed by the skin, resulting in the emission of photons that carry thermal energy [4]. Chromophores in the skin, such as hemoglobin, water, and melanin absorb photons and are then heated by thermal energy, leading to their breakdown via thermocoagulation. Unlike lasers that emit monochromatic (single wavelength), collimated (parallel waves), and coherent (waves in sync) light, IPL generates noncoherent, polychromatic light that spans a wide spectrum of wavelengths. This characteristic enables the concept of selective photothermolysis, given that different chromophores have distinct light-absorption wavelengths [5]. Beyond the use of cut-off filters, which enable the targeting of specific tissues, IPL therapy encompasses two other essential principles. The first principle concerns pulse duration, which is grounded in the thermal relaxation time (TRT) of the chromophore. To prevent thermal damage to tissues, the pulse duration should be set shorter than the chromophore’s TRT. Overheating the target beyond its TRT can lead to negative outcomes like scarring or hypopigmentation. To counteract these potential complications, many IPL machines offer delay times between consecutive pulses. These intervals typically range from 10 to 12 ms in lighter-skinned individuals, aligning with the epidermal TRT. As such, when employing IPL, clinicians must consider the specific chromophore they are targeting and then calibrate the optimal pulse duration and interval. The second fundamental principle in IPL treatment involves adjustment of the fluence. Fluence, measured in J/cm^2^, quantifies the energy density directed at the targeted area. IPL therapy can vary in intensity, starting from a modest 8 J/cm^2^ and escalating to a potent 35 J/cm^2^ in advanced settings. Deeper targets or chromophores that absorb light poorly might necessitate a higher fluence. As can be expected, side effects tend to occur more frequently at higher fluences [6].

Nevertheless, IPL-based technology is generally considered a safe procedure as potentially harmful ultraviolet radiation is typically filtered by blocking wavelengths below 500 nm. Most common adverse effects of IPL treatments have been well documented, and include blistering, hypopigmentation, hyperpigmentation, and if extensive, even scarring [7]. According to literature, no adverse effects have been documented on bone tissue.

According to recent literature, IPL has been found to possess anti-inflammatory properties through at least a dual mechanism involving the downregulation of TNF-α [8], and the upregulation of IL-10 [9,10]. TNF-α is a pro-inflammatory cytokine that plays a crucial role in the initiation and propagation of inflammatory responses. IPL therapy has been shown to suppress its expression by at least 17.6%. On the other hand, IPL treatment also leads to the upregulation of IL-10 by up to 2.74-fold. IL-10 is an anti-inflammatory cytokine that helps regulate and dampen the inflammatory process by inhibiting the induction of various inflammatory mediators, such as TNF-α, IL-1β, IL-12, and IFN-γ. Furthermore, IPL may also improve the collagen synthesis effect on cellular metabolism by affecting the electron transport chain of mitochondria [11].

Photobiomodulation (PBM) is another technology that utilizes non-ionizing light sources, primarily operating within the red to near-infrared spectrum. The wavelengths typically span from approximately 600 to 1000 nanometers (nm). This specific range is optimal for penetrating tissue and interacting with mitochondrial chromophores to stimulate cellular activity and promote healing. The therapeutic light in PBM is typically delivered at low energy levels, which do not heat the tissue, thereby avoiding thermal damage [12,13].

In contrast, intense pulsed light (IPL) technology utilizes a much broader spectrum of light wavelengths, typically from 400 to 1200 nm. IPL devices employ high-energy outputs that cause a photothermal effect, targeting specific chromophores in the skin and collagen stimulation. The intense light bursts are absorbed by the target areas, resulting in heat generation which is the primary mechanism of action in IPL treatments.

The fundamental difference lies in the application and intended outcomes: PBM aims to enhance cellular function and promote healing through non-thermal means, while IPL is used for more superficial cosmetic treatments, relying on heat to achieve its effects.

The depth to which intense pulsed light (IPL) therapy penetrates varies based on multiple factors, such as the wavelength of the light, the optical characteristics of the target tissue, and the specific IPL-device settings, including pulse duration and energy level. Generally, the penetration depth ranges from under 1 mm to several millimeters. Wavelengths between 500 and 600 nm reach the depth of small blood vessels in the skin, usually no more than 1.5 mm [14]. Given the absence of prior data on IPL penetration in gingival tissue, it is hypothesized that the penetration depth there might be approximately 1–2 mm.

Intense pulsed light (IPL) has not traditionally been used in dentistry due to its broad spectrum and high energy output. However, recent advancements and controlled studies are beginning to explore its potential applications within the oral cavity, offering promising avenues for therapeutic intervention. IPL could be effective in treating benign oral mucosal lesions like oral lichen planus or recurrent aphthous stomatitis by modulating inflammatory responses and accelerating healing. Additionally, it may play a role in managing inflammatory periodontal diseases such as gingivitis and periodontitis, through its anti-inflammatory and antimicrobial effects, potentially aiding in non-surgical treatment approaches. IPL’s capabilities extend to photobiomodulation for pain management, possibly providing relief for conditions such as temporomandibular joint disorders or post-surgical pain by promoting healing and reducing inflammation [15]. Furthermore, its use in enhancing wound healing post-extraction or surgical interventions, and treating oral dyschromia and pigmentation issues, demonstrates its versatility, echoing its successful applications in dermatology. These emerging applications suggest that IPL could become a valuable tool in dental therapies. Despite IPL’s promising anti-inflammatory effects across various domains, there are reservations regarding its application within the oral cavity due to potential thermal harm to both soft and hard tissues. Therefore, before exploring its benefits further, conducting a clinical trial to determine its safety is imperative. As far as we are aware, this study is a pioneering histological investigation into the use of IPL in oral-cavity tissues.

The aim of this study was to examine the histological response of the tissues forming the oral cavity–the teeth, the gingival tissue, the buccal mucosa, and the alveolar bone–to IPL therapy.

## 2. Materials and Methods

This study was approved by the committee for animal experiments at the Ruth and Bruce Rappaport Faculty of Medicine at the Technion (IL-1591222). All principles of safety, ethics, and professionalism were followed according to guidelines. Three adult farm pigs were included in the trial. All three animals were anesthetized with premedication: Ketamine (20 mg/kg, i.m) and Xylazine hydrochloride (1 mg/kg, i.m), followed by the induction of Propofol (4–6 mg/kg). The animals were intubated and then ventilated with 100% oxygen and Isoflurane 2%, as general anesthesia, for the duration of the procedure. Fentanyl (5 mg/kg/h) was injected for analgesia. Local anesthesia was obtained prior to IPL projection by the injection of 2% Lidocain HCl with 1:100,000 epinephrine.

Following general anesthesia, the skin of the sub-malar and mandibular regions (upper and lower jaws), and the gingiva (soft tissue adjacent to the teeth) were cleaned and prepared to ensure they were free of any debris that could interfere with the treatment. Then, the appropriate wavelength and intensity of light were selected. Intense pulsed light was projected according to the parameters that were calibrated for skin use (wavelength 500–1200 nm with full contact with the tissue, with a cut-off filter of 590 nm and 755 nm) (Table 1). IPL treatment intensity ranged from a low power of 8 J/cm^2^, and increased to a high power of up to 35 J/cm^2^ in upgraded configurations. In order to test all tissues’ responses to a variety of intense lighting, we established the following program (Table 2), in which we tested the tissues’ histological responses to an extreme power of up to 25 J/cm^2^ and 755 nm, with as low as a 5 msec interval. The IPL handpiece was then placed against the treated area, and a series of rapid pulses of light were delivered to the target area. In each animal, one quadrant of the jaw was left untreated and served as a control. Clinical assessment of the gingiva and the skin included the color of the tissue, the texture, and the existence of either ulceration or burn.

### 2.1. Histological Analysis

**Figure 1:**
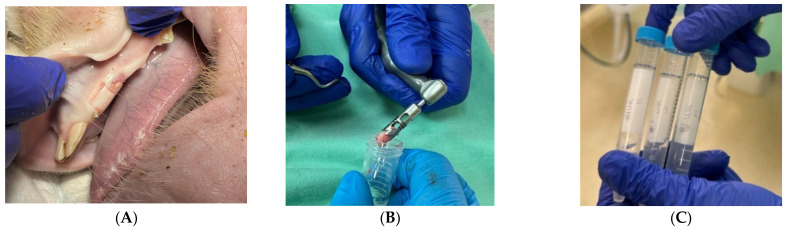
(**A**) Sample of gingival tissue taken using a 15C scalpel blade. (**B**) Sample of alveolar bone taken using a trephine 5 mm drill. (**C**) All samples were immediately immersed in 4% paraformaldehyde.

For histological analysis, tissue samples were taken from the soft tissue (keratinized tissue) on the edentulous ridge and the adjacent buccal lining mucosa, as well as from the bone underlying the covered soft tissue, which were the areas treated with IPL. Tissue samples were harvested with conventional techniques, using a 15C scalpel blade for gingiva and mucosa tissues (Figure 1A). Biopsies from the alveolar bone were obtained by a trephine 5 mm drill (Figure 1B). All sections were taken immediately after treatment or after 24 h (prior to euthanasia), as written in Table 1. All biopsies were immersed in 4% paraformaldehyde (Figure 1C) and embedded in paraffin. Gingival and mucosal tissues were cut at a thickness of 5 μm and mounted on glass slides. Hematoxylin and eosin (H&E) staining was later used to visualize the tissue architecture and evaluate the continuity of the epithelium and connective tissue, as well as to examine the possible existence of inflammation. 

Biopsies from bone were taken following decalcification with 10% EDTA solution. The samples were embedded in paraffin and cut into ~10 µm thickness sections, which were later stained with H&E to identify osteocytes and examine bone architecture. All sections were visualized and analyzed by CaseViewer^®^ (3DHISTECH Ltd. H-1141 Budapest, Öv u. 3., Hungary).

### 2.2. Animal Treatment and Handling

Post-treatment, all three animals received analgesia consisting of Tolfine (160 mg, i.m.) and Buprenorphine (0.6 mg, SC). They were fed a soft diet for the post-op day and had a veterinarian’s examination. 

Then, 24 h after the procedure, the animals were sacrificed by induction of premedication as written above, followed by an injection of Pentobarbital 200 mg/mL overdose. Subsequently, jaws were processed for histologic analysis. 

## 3. Results

### 3.1. Macroscopic Examination

An inspection of the teeth, gingiva, and mucosa was performed to assess their condition before projection, immediately after, and 24 h later. At all times, all the tissues were intact, without signs of thermal effects such as cavitation, ulceration, or burns. Moreover, inflammatory signs such as redness, swelling, or blistering were not seen. The alveolar bone bled during the drilling, as it normally should, without signs of necrosis.

### 3.2. Microscopic Examination

A total of 36 biopsies were taken, 12 from each region (gingiva, mucosa, and alveolar bone), with various set of treatments, as outlined in Table 2. Samples from every group showed clear and readable cut margins, allowing for proper histological evaluation of the samples.

#### 3.2.1. Gingiva

**Figure 2:**
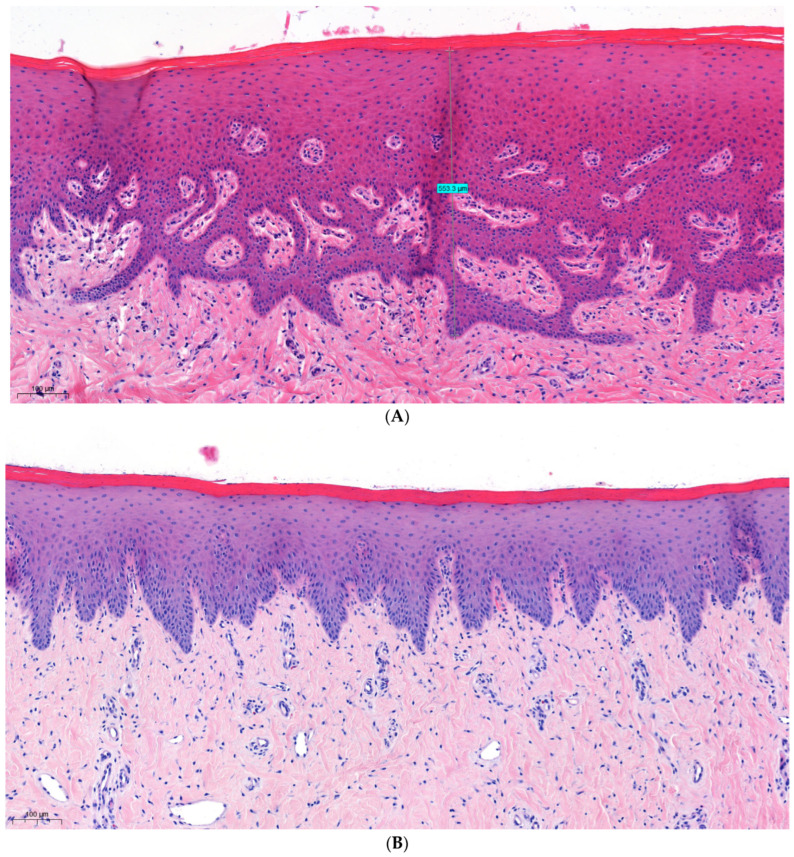

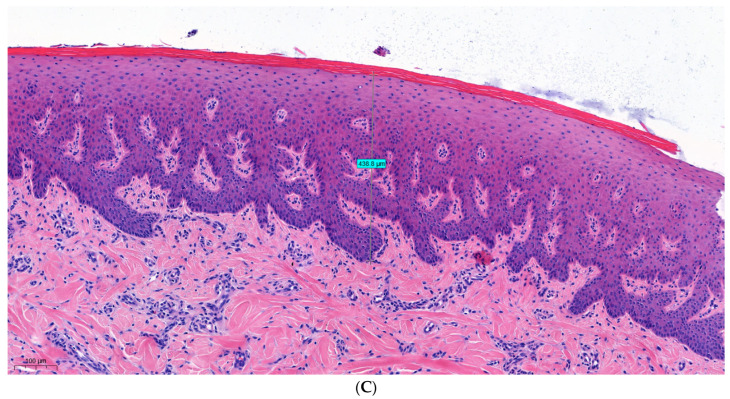
(**A**) Sample taken from the upper-left quarter of a non-treated animal, displaying normal histology of gingiva. (**B**) Gingiva sample obtained after projection with a filter of 590 nm and fluence of 20 J/cm^2^, showing no signs of thermal damage. (**C**) Gingiva sample obtained after treatment with a filter of 755 nm and fluence of 25 J/cm^2^, showing no signs of thermal damage, displaying normal oral epithelium width of 438.8 μm.

Figure 2A presents a hematoxylin and eosin slide of an upper-left gingival sample taken from a non-treated quadrant (control quadrant). The gingival tissue, as can be seen, is composed of keratinized stratified squamous epithelium tissue overlying a densely collagenous lamina propria. The epithelium facing the oral cavity constitutes the oral epithelium, while the portion facing the tooth represents the sulcular epithelium, which continues with the junctional epithelium.

A specimen from the same area, obtained after treatment with a filter of 590 nm and fluence of 20 J/cm^2^ showed no signs of peripheral thermal damage in the epithelium, nor in the lamina propria (Figure 2B). Moreover, a specimen obtained after projection with the highest intensity—a filter of 755 nm, fluence of 25 J/cm^2^ and interval of 5 ms—demonstrated a similar result, with no apparent thermal damage (Figure 2C), and a measured keratin and oral epithelium tissue width within the normal range (Table 3).

#### 3.2.2. Buccal Mucosa

**Figure 3:**
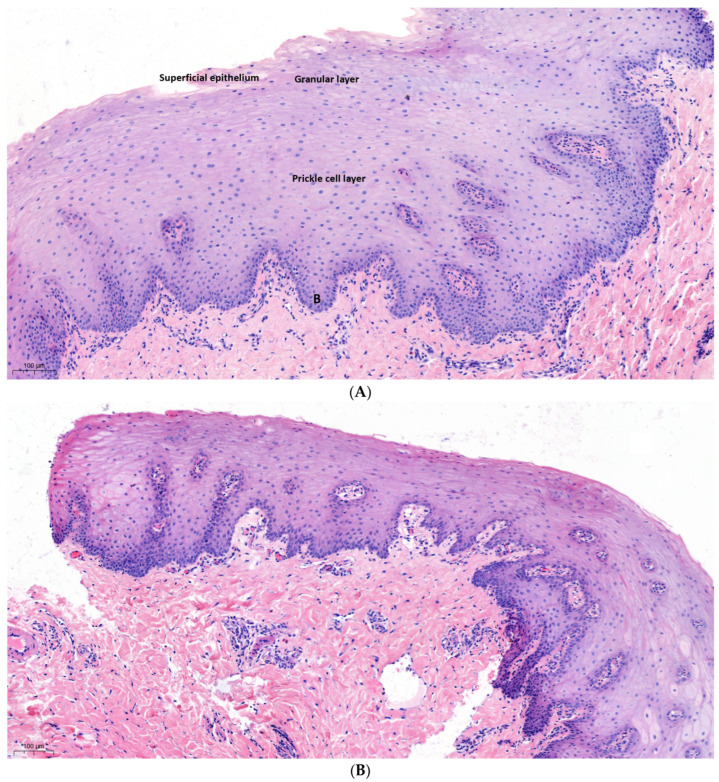

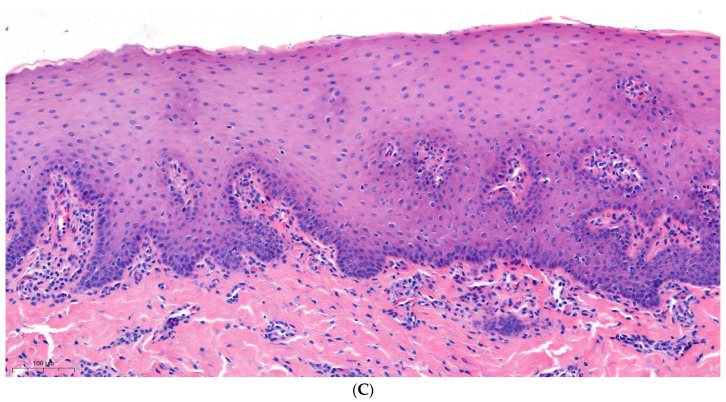
(**A**) Oral mucosa sample taken from the upper-left quarter of a non-treated quadrant, displaying normal histology. B-stratum basale (**B**) Oral mucosa sample obtained after projection with a filter of 590 nm and fluence of 20 J/cm^2^, showing no signs of thermal damage. (**C**) Oral mucosa sample obtained after treatment with a filter of 755 nm, fluence of 25 J/cm^2^, and interval of 5 ms, showing no signs of thermal damage.

The oral mucosa is the wet soft tissue membrane lining the structures within the oral cavity. Histologically, it is formed by stratified, non-keratinized squamous epithelium, displaying four distinct layers: the superficial epithelium, the intermediate layer, the prickle cell layer, and the mitotically active basal cells (B), which reside on the basement membrane, separating the epithelium from the underlying lamina propria (Figure 3A).

Similar to gingiva, the specimen obtained after using a filter of 590 nm and fluence of 20 J/cm^2^ showed no signs of thermal damage or inflammation, and histology remained normal, with no loss of cellular or tissue architecture. (Figure 3B). Moreover, the specimen obtained after projection with the highest intensity—a filter of 755 nm, fluence of 25 J/cm^2^, and interval of 5 ms—demonstrated a similar result, with no presentation of thermal damage (Figure 3C). Also, clinically, no adverse effects such as redness, swelling, or blistering of the buccal mucosa were visible.

#### 3.2.3. Alveolar bone

**Figure 4:**
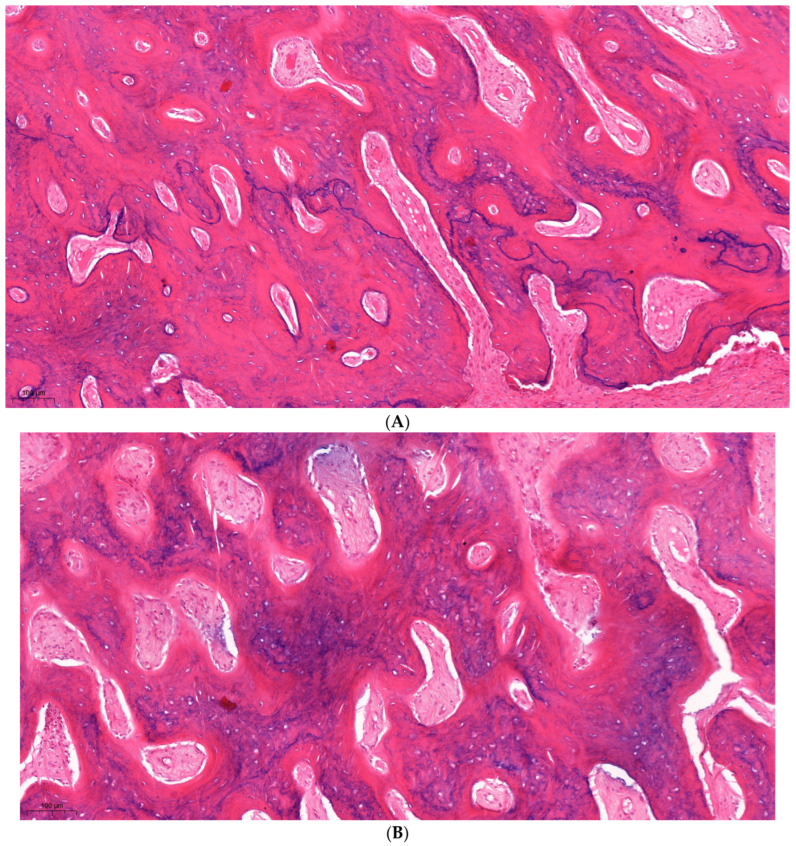

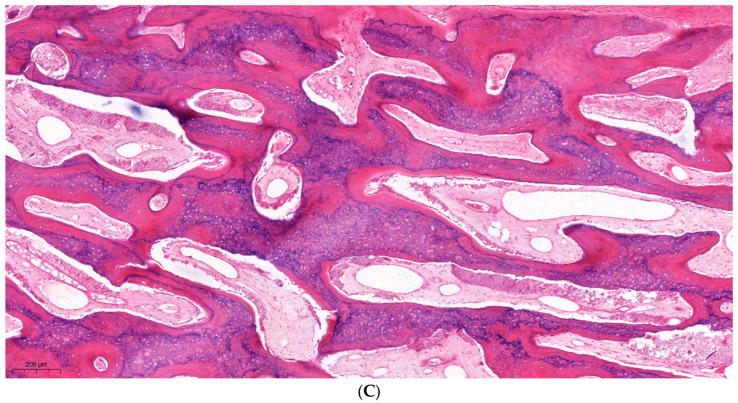
(**A**) Alveolar bone sample taken from the upper-left quadrant of a non-treated quadrant, displaying normal histology. (**B**) Alveolar bone sample obtained after projection with a filter of 590 nm and fluence of 20 J/cm^2^. (**C**) Alveolar bone sample obtained after projection with a filter of 755 nm, fluence of 25 J/cm^2^ and interval of 5 ms, showing no signs of thermal damage or changes in cell characterization.

Alveolar processes of the maxilla and the mandible are one of the supporting tissues of the teeth, containing the sockets for the roots of the teeth. Histologically, they consist of a compact bone, which forms the outer and inner plates of the alveolar process, and a spongy bone, which fills the space between the alveolar bone proper and cortical plates. Osteocytes were identified as round or oval-shaped cells within the lacunae, surrounded by mineralized bone matrix. Figure 4A displays a section obtained from a control quadrant, showing normal bone histology.

Despite the high intensity of the light energy used, histology sections performed after the treatment showed that the alveolar bone remained unharmed. Figure 4B,C shows samples obtained after projection with a filter of 590 nm and 755 nm, respectively. Moreover, osteocyte number density was determined by estimating the number of osteocytes per unit area (#osteocytes/mm^2^) in randomly selected microscopic fields using a light microscope at a magnification of 400×, and showed similar results (Table 4).

## 4. Discussion

Over time, studies have been carried out to quantify the portion of tissue damaged by heat produced by light, both in vivo and ex vivo, using different kinds of light-emitting modalities. Studies examining the thermal damage caused by lasers were conducted by Romeo et al. using different wavelengths, and concluded that all types of lasers tested (diode 980 nm, diode 808 nm, diode 445 nm, Nd:YAG, Er,Cr:YSGG, KTP, and CO_2_) can be used to perform biopsies if used properly. Even when cut edges showed some thermal damage, a histological diagnosis could have been made [16,17]. Monteiro et al. came to a similar conclusion in a study in which they analyzed the resection margins of fibroepithelial lesions obtained using CO_2_ lasers, diode lasers, Er:YAG lasers, and Nd:YAG lasers in vivo compared with the electrosurgical scalpel and cold scalpel, and they assessed that none of the tested lasers showed limitations regarding histological diagnosis [18]. It is important to note that every type of laser creates thermal damage to the target tissues to some extent through a photothermal effect. The laser beam transmits thermal energy that increases the temperature at the point of incidence by more than 100 °C The increase in the temperature of the surrounding tissues can cause permanent or reversible damage. The extent of the damage is related to both the wavelength and the setting of the laser.

Intense pulsed light (IPL) systems are high-intensity light sources that, unlike laser systems, emit polychromatic light. Their flashlamps work with noncoherent light in a broad wavelength spectrum of 515–1200 nm. Moreover, settings like fluence, pulse rate, and interval can be adjusted to determine the intensity of IPL therapy. These properties allow for great variability in selecting individual treatment parameters and adapting them to different types of tissues and indications. IPL-based technology is generally considered a safe procedure, as potentially harmful ultraviolet radiation is typically filtered by blocking wavelengths under 500 nm.

The use of IPL technology has been shown to be a promising approach for various procedures. A prior investigation employing the same IPL filters—PR (530–750 nm) and VL (555–950 nm)—on patients with microstomia and systemic sclerosis revealed significant outcomes. The participants underwent eight treatments in the perioral area at intervals of 3–4 weeks, with a follow-up period of 6 months. The study reported a notable increase in the inter-ridge distance, with the mouth opening expanding by 4.1 mm (95% confidence interval, 1726–6638, *p* < 0.005), comparing measurements from before the treatment to those at the 6-month follow-up [19].

IPL technology presents a promising technique for various applications. Yet, reservations have been raised concerning potential thermal harm to both the soft and hard tissues within the oral cavity. This research endeavored to analyze the histological reaction of oral-cavity tissues when exposed to IPL. To our understanding, this is a pioneering histological study of IPL’s impact on oral tissues. For our analysis, we employed pig intra-oral tissues. Given their anatomical and physiological resemblance to human tissues, predictable patterns in wound healing and injury responses, and ethical considerations, pig models can provide valuable insights into the safety and efficacy of IPL for various dental applications [20].

We found that there was no significant thermal damage to the soft and hard tissues forming the oral cavity. Our results suggest that the use of IPL in the oral cavity is safe and does not negatively affect the tissues examined. Moreover, no changes in the characterization of the cells were observed when compared to the control group. These findings are consistent with previous studies that have reported minimal thermal damage associated with the use of IPL. The lack of thermal damage observed in our study may be attributed to the controlled application of IPL and the use of appropriate settings that ensured safe and effective treatment. Overall, our study provides valuable insights into the safety of IPL in the oral cavity and may help inform the development of future dental procedures that utilize this technology.

The scattering phenomenon in the oral cavity can significantly affect the efficacy of IPL treatment by altering the power distribution of the light as it penetrates the tissues. This scattering can lead to a reduction in the intensity of light reaching deeper tissues, potentially diminishing the therapeutic effects of IPL. According to our search, this issue has not been discussed extensively in the literature concerning oral-cavity tissues and should be addressed in future studies to optimize IPL applications in dental procedures.

It is important to mention that the current IPL handpiece is not applicable in the human oral cavity. Therefore, should positive effects be observed, there will arise a need to design, adjust, and tailor an appropriate device for human application. Additionally, establishing a comprehensive protocol for each specific condition will be essential to optimize results and minimize potential risks or complications.

Limitations of this study related primarily to the efficacy of testing. As this research was an initial study focused mainly on examining the safety of IPL use in the oral cavity, no beneficial effects could be documented. Also, the clinical examination of potential side effects was restricted to a 24 h timeframe to mitigate extended discomfort for the treated animals. Another potential limitation to consider is the long-term effects of this technology on tissues, particularly how they might be impacted if the duration of IPL exposure is extended. Additionally, the effects on tooth vitality were not tested due to the setup of our research; however, we did not observe any external changes in the teeth, such as color changes or alterations in the structure of the enamel.

It is evident that only tangible and visible adverse effects were recorded, as there was no feasible method to capture any subjective symptoms experienced by the animals.

## 5. Conclusions

This study showed that the IPL can be safely used during oral procedures, with controlled power settings and fluence. Further studies, especially in vivo investigations, are needed to confirm these results.

## Figures and Tables

**Table 1:** The division of the study groups according to the oral cavity quadrant. Three biopsies were obtained from each quadrant, either immediately after treatment (0 h) or after 24 h.

	Quadrant			
Pig	UR	UL	LR	LL
#1	Treatment: IPL Biopsies: 1 × mucosa (0 h) 1 × gingiva (0 h) 1 × bone (0 h)	Treatment: IPL Biopsies: 1 × mucosa (24 h) 1 × gingiva (24 h) 1 × bone (24 h)	Treatment: IPL Biopsies: 1 × mucosa (0 h) 1 × gingiva (0 h) 1 × bone (0 h)	Treatment: Control Biopsies: 1 × mucosa (24 h) 1 × gingiva (24 h) 1 × bone (24 h)
#2	Treatment: IPL Biopsies: 1 × mucosa (24 h) 1 × gingiva (24 h) 1 × bone (24 h)	Treatment: Control Biopsies: 1 × mucosa (0 h) 1 × gingiva (0 h) 1 ×bone (0 h)	Treatment: IPL Biopsies: 1 × mucosa (24 h) 1 × gingiva (24 h) 1 × bone (24 h)	Treatment: IPL Biopsies: 1 × mucosa (0 h) 1 × gingiva (0 h) 1 × bone (0 h)
#3	Treatment: Control Biopsies: 1 × mucosa (0 h) 1 × gingiva (0 h) 1 × bone (0 h)	Treatment: IPL Biopsies: 1 × mucosa (24 h) 1 × gingiva (24 h) 1 × bone (24 h)	Treatment: IPL Biopsies: 1 × mucosa (24 h) 1 × gingiva (24 h) 1 × bone (24 h)	Treatment: IPL Biopsies: 1 × mucosa (0 h) 1 × gingiva (0 h) 1 × bone (0 h)

UR—upper right, UL—upper left, LR—lower right, LL—lower left.

**Table 2:** Settings of filter, fluence, pulse duration, and interval that were used, according to the different quadrants.

Pig	#1				#2				#3			
Quadrant	UL	UR	LL	LR	UL	UR	LL	LR	UL	UR	LL	LR
Filter (nm)	590	590	CTR	755	CTR	755	590	755	590	CTR	755	755
Fluence (J/mc^2^)	20	16		20		16	20	20	20		25	25
Pulse duration (msec)	6	6		6		3	3	3	3		3	3
Interval (msec)	50	50		50		10	10	10	5		5	5

UR—upper right, UL—upper left, LR—lower right, LL—lower left.

**Table 3:** Measurement of keratin and oral epithelium thickness (μm) of the gingival tissue after projection in various energy settings.

Filter (nm)	Fluence (J/mc^2^)	Pulse Duration (msec)	Interval (msec)	Keratin Thickness (μm)	Oral Epithelium Thickness (μm)
Control				27.2	553.3
590	20	6	50	24.2	432.2
755	25	3	5	27.6	438.8

**Table 4:** Estimated osteocyte number density, after projection with various set of intensities.

Filter (nm)	Fluence (J/mc^2^)	Pulse Duration (msec)	Interval (msec)	Osteocytes per Determined Unit Area
Control				41
590	20	6	50	45
755	25	3	5	42

## Data Availability

The data presented in this study are available on reasonable request from the corresponding author.
